# Interspecific Variation in Life History Relates to Antipredator Decisions by Marine Mesopredators on Temperate Reefs

**DOI:** 10.1371/journal.pone.0040083

**Published:** 2012-06-29

**Authors:** Alejandro Frid, Jeff Marliave, Michael R. Heithaus

**Affiliations:** 1 Vancouver Aquarium, Vancouver, British Columbia, Canada; 2 Department of Biological Sciences, School of Environment, Arts, and Society, Florida International University, North Miami, Florida, United States of America; University of Alberta, Canada

## Abstract

As upper-level predatory fishes become overfished, mesopredators rise to become the new ‘top’ predators of over-exploited marine communities. To gain insight into ensuing mechanisms that might alter indirect species interactions, we examined how behavioural responses to an upper-level predatory fish might differ between mesopredator species with different life histories. In rocky reefs of the northeast Pacific Ocean, adult lingcod (*Ophiodon elongatus*) are upper-level predators that use a sit-and-wait hunting mode. Reef mesopredators that are prey to adult lingcod include kelp greenling (*Hexagrammos decagrammus*), younger lingcod, copper rockfish (*Sebastes caurinus*) and quillback rockfish (*S. maliger*). Across these mesopredators species, longevity and age at maturity increases and, consequently, the annual proportion of lifetime reproductive output decreases in the order just listed. Therefore, we hypothesized that the level of risk taken to acquire resources would vary interspecifically in that same order. During field experiments we manipulated predation risk with a model adult lingcod and used fixed video cameras to quantify interactions between mesopredators and tethered prey (*Pandalus* shrimps). We predicted that the probabilities of inspecting and attacking tethered prey would rank from highest to lowest and the timing of these behaviours would rank from earliest to latest as follows: kelp greenling, lingcod, copper rockfish, and quillback rockfish. We also predicted that responses to the model lingcod, such as avoidance of interactions with tethered prey, would rank from weakest to strongest in the same order. Results were consistent with our predictions suggesting that, despite occupying similar trophic levels, longer-lived mesopredators with late maturity have stronger antipredator responses and therefore experience lower foraging rates in the presence of predators than mesopredators with faster life histories. The corollary is that the fishery removal of top predators, which relaxes predation risk, could potentially lead to stronger increases in foraging rates for mesopredators with slower life histories.

## Introduction

Overfishing has caused the global decline of upper-level predatory fishes [1]. Consequently, in many marine communities prey of overfished predators have increased numerically and may have altered their behaviour in response to relaxed predation pressure [2], [3]. As expected from classic theory on top-down control [4], herbivores released from predation have flourished and increased their impact on plants [2], [5]. Perhaps more notably, predators that previously occupied mid-trophic levels have risen from their former mesopredator status to becoming the new top predators of over-exploited marine communities, often contributing to shifts in ecosystem processes [2], [5]. The ‘rise of the mesopredator’ and its implications for food web structure is increasingly recognized as a general conservation problem across marine, terrestrial and freshwater communities [6].

Life history theory and the existing body of work on antipredator behaviour provide a basis for predicting some changes that marine food webs might undergo as top predators become overfished and mesopredators rise to the top of ‘flattened trophic pyramids’ (a term coined by Strong and Frank [2]). In addition to affecting prey density through consumption, predators induce prey to engage in antipredator behaviours–such as vigilance, use of refuges, and avoidance of dangerous patches–that have the cost of reducing access to resources [7]. Prey antipredator behaviour, therefore, may mediate some indirect effects of top predators to lower trophic levels [8].

Less known is the extent to which prey species with similar trophic levels but contrasting life histories differ in their willingness to risk predation to acquire resources. Among iteroparous species that reproduce annually, the annual proportion of lifetime reproductive output is lower for long-lived species with late maturity than for shorter-lived species that mature earlier. Within the latter species, individuals might maximize fitness by taking high risks to acquire food, mates, or other resources that enhance short-term reproductive success. In contrast, individuals of species with longer lives and later maturity might maximize fitness by being more averse of predation risk, even at the cost of reduced access to resources, thereby enhancing their chances of survival and reproduction into late adulthood [9], [10], [11].

We conducted field experiments with tethered prey and a model predator to test the hypothesis that life history characteristics affect the level of risk taken by marine mesopredators to acquire resources. Our study took place in temperate reefs of the northeast Pacific Ocean, where adult lingcod (*Ophiodon elongatus*) are upper-trophic level predators that use a sit-and-wait hunting mode, while kelp greenling (*Hexagrammos decagrammus*), younger lingcod, copper rockfish (*Sebastes caurinus*) and quillback rockfish (*S. maliger*) are common mesopredators [12], [13] that are prey of adult lingcod [14], [15]. These mesopredators share habitats and many diet items, including demersal shrimps of the genus *Pandalus* [13], [14], [16] and represent a broad range of life history characteristics. Kelp greenling have the fastest life history; their maximum age is 12 to 13 years and age at maturity is 3 to 5 years [17], [18]. Lingcod have a slightly slower life history than kelp greenling; although their age at maturity (2 years for males, 3 to 5 years for females) is similar to that of kelp greenling, their maximum lifespan is longer (14 to 16 years for males, 20 years for females) [18], [19]. Copper rockfish have a much slower life history than kelp greenling and lingcod; they live up to 50 years and in British Columbia age at 50% maturity is six to seven years [12]. Quillback rockfish have the slowest life history; they live up to 95 years and in British Columbia females reach 50% and 100% maturity at 11 and 22 years of age, respectively [12]. Notably, offspring quality (i.e. larval oil globule volume) and fecundity are positively related to maternal age of rockfish, underscoring the importance of long term survival to fitness for this genus [12], [20].

We predicted that (1) attack and inspection probabilities would rank from highest to lowest and the timing of attacks during a trial would rank from earliest to latest in the following order: kelp greenling, subadult lingcod, copper rockfish, and quillback rockfish. We also predicted that (2) responses to a large model lingcod, such as avoidance of interactions with tethered prey, would rank from weakest to strongest in the same order.

## Methods

### Ethics Statement

Florida International University approved our project and issued permit number 11-035 under its Institutional Animal Care and Use Committee Protocol. The Vancouver Aquarium approved our SCUBA-based fieldwork via a permit issued by the Vancouver Aquarium Dive Safety Officer. The Vancouver Aquarium did not require Animal Care and Use Committee approval for our study because we did not capture or otherwise handle vertebrates. All field work took place in public areas where SCUBA diving is permitted.

**Figure 1:**
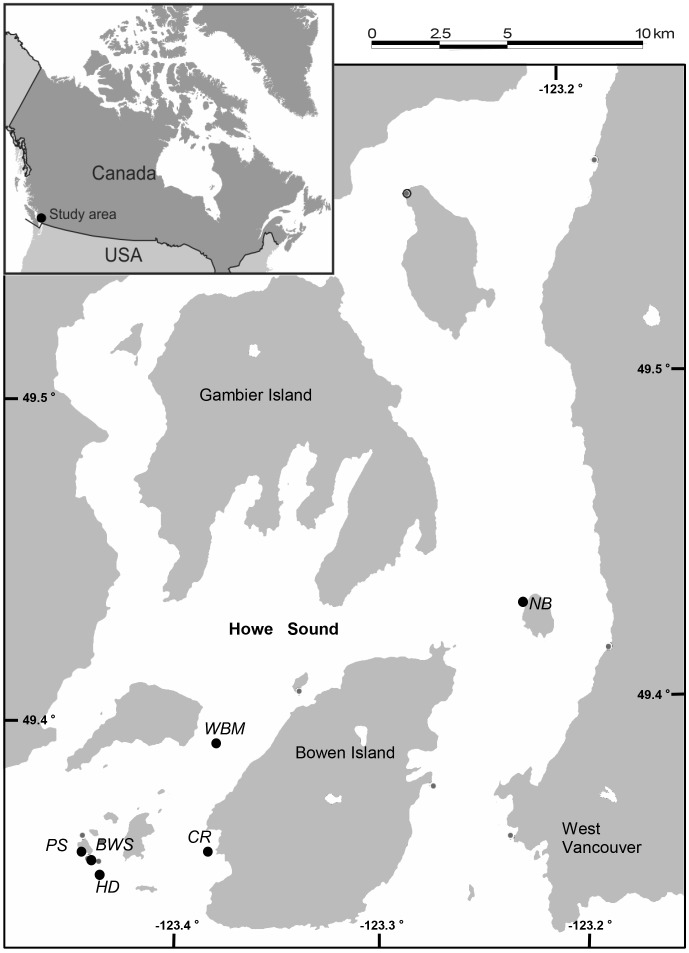
Map of the study area. Black circles represent study reefs (labelled in italics).

SCUBA-based fieldwork took place at six reefs of Howe Sound, British Columbia, Canada (Fig. 1), between 7 October and 7 December 2011. Reefs ranged in depth from 8 to 16 m below mean low tide.

We used tethered prey experiments to determine foraging and antipredator decisions by different species of mesopredators. Live *Pandalus* shrimps for tethering were acquired outside the study reefs; they were much larger than resident shrimp (total length 10 to 14 cm, compared to ≤4 cm), and assumed to be a major reward for fish. Shrimps were tethered with 10 to 20 cm-long monofilament fishing line (2 1b test) looped around their torso at one end and attached by the opposite end to a 1 m-long chain. Three shrimps were attached 20 cm apart to each chain. The exceptions were 3 chains (of 22 total) with 1 or 2 shrimps. Video cameras (GoPro, Woodman Labs Inc.) placed within 90 cm of tethered shrimps recorded mesopredator identity and the timing of interactions between mesopredators and tethered prey. To achieve unobstructed camera views, tethered shrimp and cameras were placed on flat bottom immediately adjacent (≤1.5 m away) to the structurally-complex boulder habitats preferred by reef fishes.

**Figure 2:**
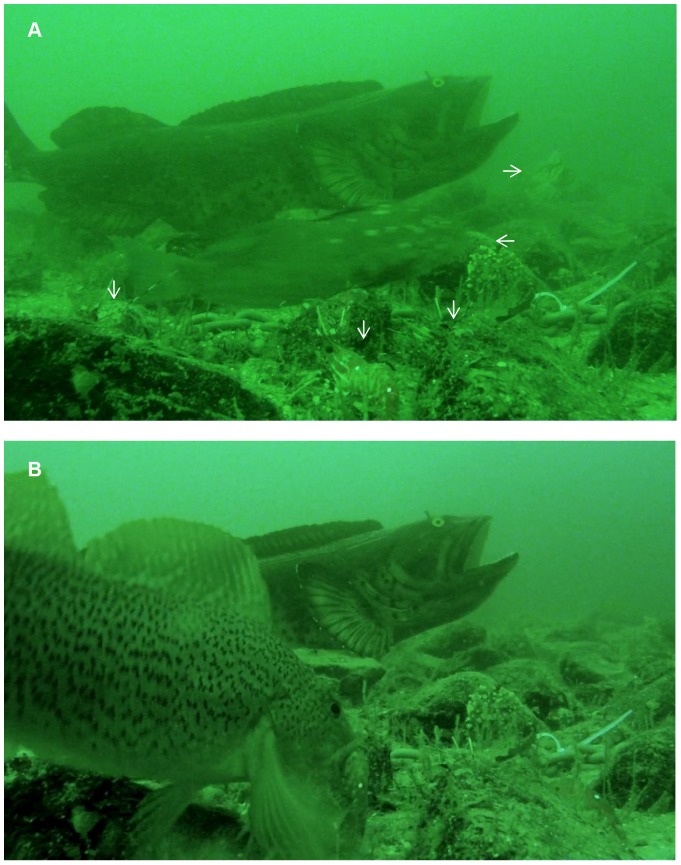
Interactions between mesopredators and tethered prey adjacent to the model predator (fibreglass replica of an adult lingcod seen in the background). In panel A vertical arrows point to *Pandalus* shrimps tethered to the chain behind (only antennae are visible for peripheral shrimps). The left-pointing arrow indicates a male kelp greenling closely inspecting prey while swimming rapidly through the vicinity of the model predator. The right-pointing arrow indicates a copper rockfish inspecting prey while slowly swimming at a greater distance. Panel B shows an attack by a female kelp greenling, the species least responsive to the model predator, during the same trial.

Experimental manipulations were as follows. The ‘adjacent predator’ treatment was spatially replicated on four reefs (CR, BWS, HD, and WBM: See Fig. 1); it consisted of a 125 cm-long model of an adult lingcod (fibreglass taxidermic casting) placed within 75 cm of the center of a chain with tethered shrimps (Fig 2). We assumed that this model predator would alter fish behaviour reef-wide but, given the sit-and-wait hunting mode used by lingcod, its effects would be strongest within a radius of 5 m. Accordingly, the same trials involving the model predator also included a ‘distant predator’ treatment; it consisted of two chains with tethered shrimp placed 8 m to 20 m from the model predator. The ‘no predator’ treatment was spatially replicated on four reefs and consisted of two or three chains with tethered shrimps (reefs PS and CR vs. BWS and NB, respectively) placed 8–20 m apart. We assumed that this treatment would measure species differences in willingness to exploit a novel resource, a trait that may correlate with willingness to incur greater predation risk [21]. Two reefs (CR and BWS) were used for the no predator treatment and, two weeks later, for trials involving the model predator; the remaining four reefs were used only for one treatment type.

After setting up the experiments, divers left the reef for up to 4 hours (the battery life of cameras). Upon return, divers counted and estimated the sizes of fish along a standardized transect (30 m long×4 m wide). A ruler attached to the end of a pole was used to estimate fish sizes. These counts covered the structurally complex habitats that were the point of origin for mesopredators interacting with tethered prey. Divers retrieved materials and cameras after these counts.

Behaviours scored during video analyses were as follows. *Inspection* consisted of head orientation towards tethered prey and associated with either a reduction in swimming speed, a change from swimming to resting on the bottom or, if swimming rapidly across the video frame, approaching within five body lengths of tethered prey (Fig. 2a). *Attack* consisted of a directed approach towards individual prey culminating with the placement of prey inside the mesopredator’s mouth (Fig. 2b). To avoid artificially inflating a species’ apparent attack rate, we considered attacks by the same species to be independent only if they were spaced apart by ≥5 min. This threshold was based on data showing that when the same species of mesopredator (namely kelp greenling) attacked multiple prey during the same trial of a given treatment, most attacks occurred either within 3 min of each other or were spaced apart by at least 5 min (range = 5.5 to 73 min: Fig. S1). If multiple attacks were non-independent, then only the first attack was scored for analysis.

To account for the effects of local species densities (Fig S2), the relative probability of behaviour *B* occurring during a trial was calculated for mesopredator species *s* during treatment *t* at reef *r* as:
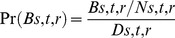
(1)where *D* is the local density of the mesopredator species. If the behaviour is inspection, then *B* is the number of inspections and *N* is the number of chains with tethered prey available to inspect (i.e., we assumed that all shrimps on a chain were inspected simultaneously). If the behaviour is attack, then *B* is the number of independent attacks and *N* is the number of available prey (both summed for all chains of treatment *t* in reef *r*) at the time of attack. That is, at the start of a trial all mesopredator species have the same prey base available. After an attack by species *s*, however, the prey base available to the remaining species is recalculated by subtracting the number of shrimp consumed.

Data did not meet normality assumptions and were analyzed with non-parametric statistics [22] using SYSTAT 13.

## Results

Attacks by mesopredators occurred at 86% of chains with tethered prey (N = 22) and 71% of individual shrimps (N = 62) were consumed. From reference points within the video images (e.g. size of chain links or model lingcod), we estimated that most rockfish and kelp greenling interacting with prey were of adult size (total length ≥20 cm) while most lingcod were subadults (total length ≤50 cm).

**Figure 3:**
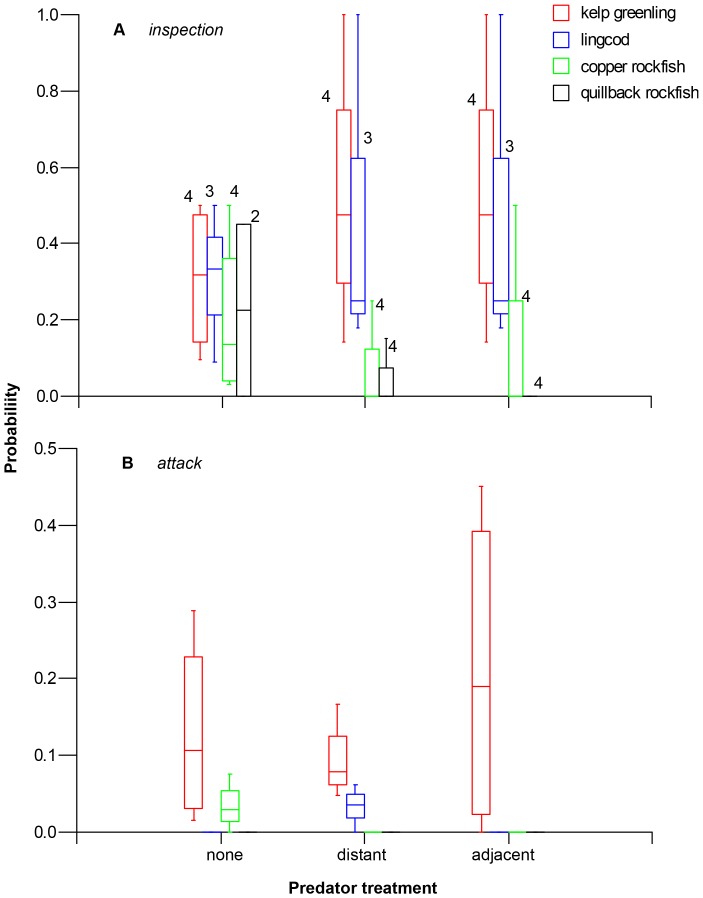
Box plots comparing the probabilities that different species of mesopredators will (A) inspect or (B) attack tethered prey during experimental treatments. Numbers above boxes in panel A indicate sample sizes (i.e., number of reefs in which the treatment was replicated and the particular species was present); these same numbers apply to panel B. Boxes enclose the median (centerline) and 25th and 75^th^ percentiles (boundaries of the box); line caps indicate 10th and 90th percentiles.

In the absence of the model predator, inspection probabilities were similar across mesopredator species (Kruskal-Wallis Test Statistic  = 1.22, P = 0.75; Fig. 3a). Species differences, however, were evident in the presence of the model predator (both distant and adjacent to prey treatments: Kruskal-Wallis Test Statistics ≥8.66, P≤0.034), when kelp greenling and lingcod were more likely to inspect prey than copper and quillback rockfish (Post hoc pairwise comparisons: Conover-Inman statistics ≥2.64, P≤0.023; Fig. 3a), Inspection probabilities in the presence of the model predator, however, did not differ between rockfish species (Conover-Inman statistic = 0.29, P = 0.78) or between kelp greenling and lingcod (Conover-Inman statistic = 0.13, P = 0.90) (Fig. 3a). Both rockfish species tended to conduct less inspections when the model predator was present (Fig. 3a), but statistical support for this relationship was weak (All Kruskal-Wallis test statistics ≤3.0, DF = 2. P≥0.223).

Attack probabilities were higher for kelp greenling than for other species during all experimental treatments (Kruskal-Wallis Test Statistics ≥8.23, P≤0.041; Fig. 3b). This result was strongly supported during treatments involving the model predator (Conover-Inman statistics ≥3.13, P≤0.010). In the absence of the model predator, however, the difference between kelp greenling and copper rockfish was statistically weak (Conover-Inman statistic = 1.56, P = 0.15).

**Figure 4:**
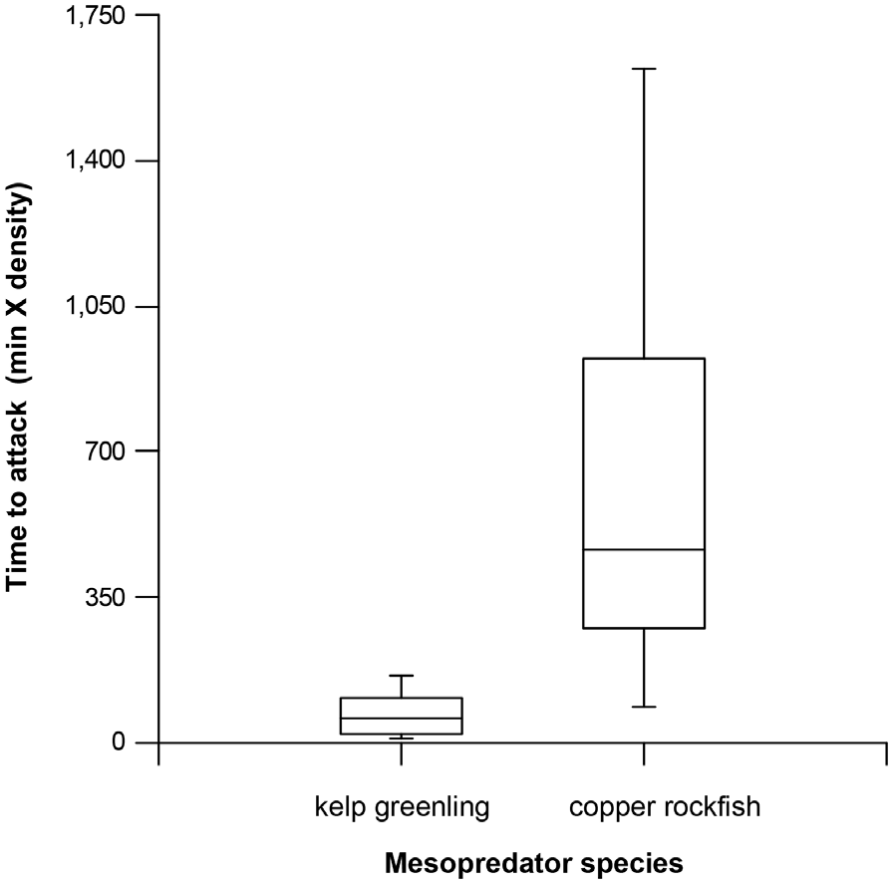
Box plots comparing times to attack by kelp greenling and copper rockfish in the absence of the model predator (‘no predator’ treatment). Because mesopredator densities varied by species and reef, times were multiplied by local density as a weighting factor.

The model predator did not affect the probability of attack by kelp greenling (Kruskal-Wallis Test Statistic  = 0.24, P = 0.87; Fig. 3b). The model predator, however, strongly affected copper rockfish, which attacked prey only in its absence (Kruskal-Wallis Test Statistic  = 7.16, P = 0.028; Fig. 3b). These attacks by copper rockfish (N = 6) lagged behind those conducted by kelp greenling (N = 9) during the same treatment (U = 54.0, P = 0.001; Fig. 4). Lingcod conducted only two attacks; these occurred during the distant predator treatment and lagged behind kelp greenling attacks on the same treatment by 22 to 184 min (times are weighted by local species density). Quillback rockfish never attacked prey (Fig. 3b).

## Discussion

Our results suggest that life history characteristics influence the level of risk different species of mesopredators take to acquire resources on temperate rocky reefs. As far as we are aware, our study is novel because other empirical studies, rather than comparing behaviour between different species that occupy similar trophic levels, have examined relationships between antipredator behaviour and intraspecific variation in life history characteristics [10],[23].

Kelp greenling, the species with the fastest life history, took the highest risks. Kelp greenling had the highest probability of attacking tethered prey during all treatments and were the only mesopredator to attack prey adjacent to the model predator. Lingcod have a slightly slower life history and individuals of subadult size were second to kelp greenling in risk-taking. They were the only mesopredator, other than kelp greenling, to attack prey during the distant predator treatment and had a higher probability of inspecting prey than copper and quillback rockfish when the model predator was present. Copper rockfish, which have a much slower life history than kelp greenling and lingcod, ranked third in risk-taking. They attacked prey, but only in the absence of the model predator and these attacks occurred later during trials than those of kelp greenling. Quillback rockfish have the slowest life history and took the least risks; they inspected prey only when the model predator was absent or distant and did not attack prey.

These results are consistent with our hypothesis, yet alternative explanations are plausible, such as species differences in their preference for *Pandalus* shrimps or interspecific variation in diel timing of their feeding. Diet studies are lacking for Howe Sound, and therefore neither possibility can be assessed rigorously. The available evidence, however, suggests that all four mesopredators consume *Pandalus* shrimps when these are available [13], [14], [16]. Evidence also suggests that copper and quillback rockfish may prefer to feed crepuscularly and diurnally [16], respectively, which biased our diurnal study towards observing greater foraging rates by quillback rockfish than by copper rockfish. Our conclusion that copper rockfish are more willing to take risks while foraging than quillback rockfish, therefore, is conservative.

**Figure 5:**
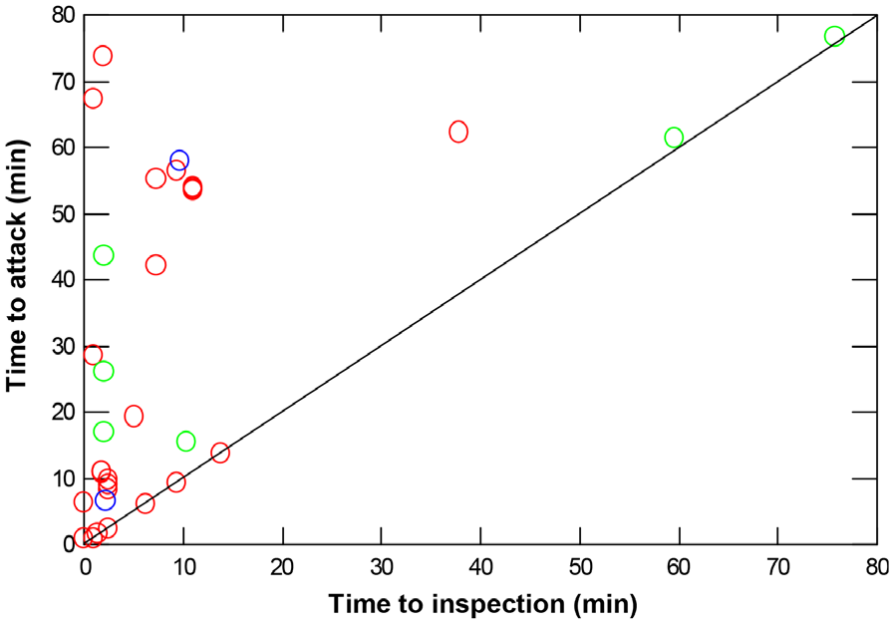
Relationship between timing of inspection of tethered prey and an attack on the same prey during a trial. The diagonal line indicates a slope value of 1. Red, blue and green symbols represent, respectively, kelp greenling, lingcod, and copper rockfish. Given that the comparisons of interest are within species, times are not weighted by local species density.

An additional alternative hypothesis is that at least some of our results were driven by species differences in their ability to gather information for locating resources (a perceptual constraint) rather than antipredator behaviour (a set of decisions). Times to inspection, however, generally were shorter than times to attack. Of 32 independent attacks on tethered shrimps, 69% occurred 5 to 72 minutes after the species conducting the attack had inspected the same prey (Fig. 5). These delays suggest that our data reflect antipredator decisions primarily.

Our study is a first step towards assessing how marine reef mesopredators with different life histories respond to predation risk. In spite of occupying similar trophic levels, short-lived species with early maturity, such as kelp greenling, and longer-lived species with later maturity, such as copper and quillback rockfish, may not be functionally redundant if differences in their antipredator behaviour affect the extent to which they transmit indirect species interactions initiated by lingcod [3], [24]. Our observations also support the notion that smaller size classes of lingcod, the ones that interacted with tethered prey, may be best understood as functional mesopredators rather than as upper level predators.

We suggest that our study has the following implications for predicting ecological change in over-exploited reef communities. First, fishing, which tends to skew the size and age structure of predatory fishes towards smaller, younger fish [1], could suppress the capacity for young lingcod to reach a larger size and higher trophic level. Under this scenario, both rockfish and kelp greenling of adult sizes may be released from predation risk, but the potential contribution of each species to trophic cascades [24] or other indirect species interactions may not change in parallel [3]. Specifically, we predict that indirect interactions between species initiated by large lingcod are transmitted primarily by mesopredator species with slow life histories, which invest more in safety and thus experience greater reductions in foraging rates in the presence of predators than mesopredators with faster life histories. Declines in large lingcod, therefore could potentially result in greater mortality and lower foraging rates for invertebrates primarily due to changes in rockfish behaviour. Additionally, lingcod and rockfish often are overfished [12], [25] while fisheries target kelp greenling less intensely. These conditions could accelerate the potential rise of kelp greenling to the top of ‘flattened trophic pyramids’ [2], a scenario analogous to exploited terrestrial communities where former top predators like wolves (*Canis lupus*) are being replaced by mesopredators with fast life histories like coyotes (*Canis latrans*) [6].

## Supporting Information

Figure S1 **Distribution of time intervals (min) between repeated attacks conducted by kelp greenling or copper during the same of trial.** Other species did not conduct repeated attacks.
(TIF)

Figure S2 **Fish densities estimated from counts of fish along 30 m×4 m transects conducted at the end of each experimental trial in 6 reefs of Howe Sound, British Columbia, October-December 2011. BWS and CR are the only reefs where both no predator and predator treatments occurred.**
(TIF)
