# Supplementary figures and images for: Interspecific Variation in Life History Relates to Antipredator Decisions by Marine Mesopredators on Temperate Reefs
Source: PLoS One. 2012 Jun 29;7(6):e40083. doi: 10.1371/journal.pone.0040083 (PMC3386963; doi:10.1371/journal.pone.0040083)

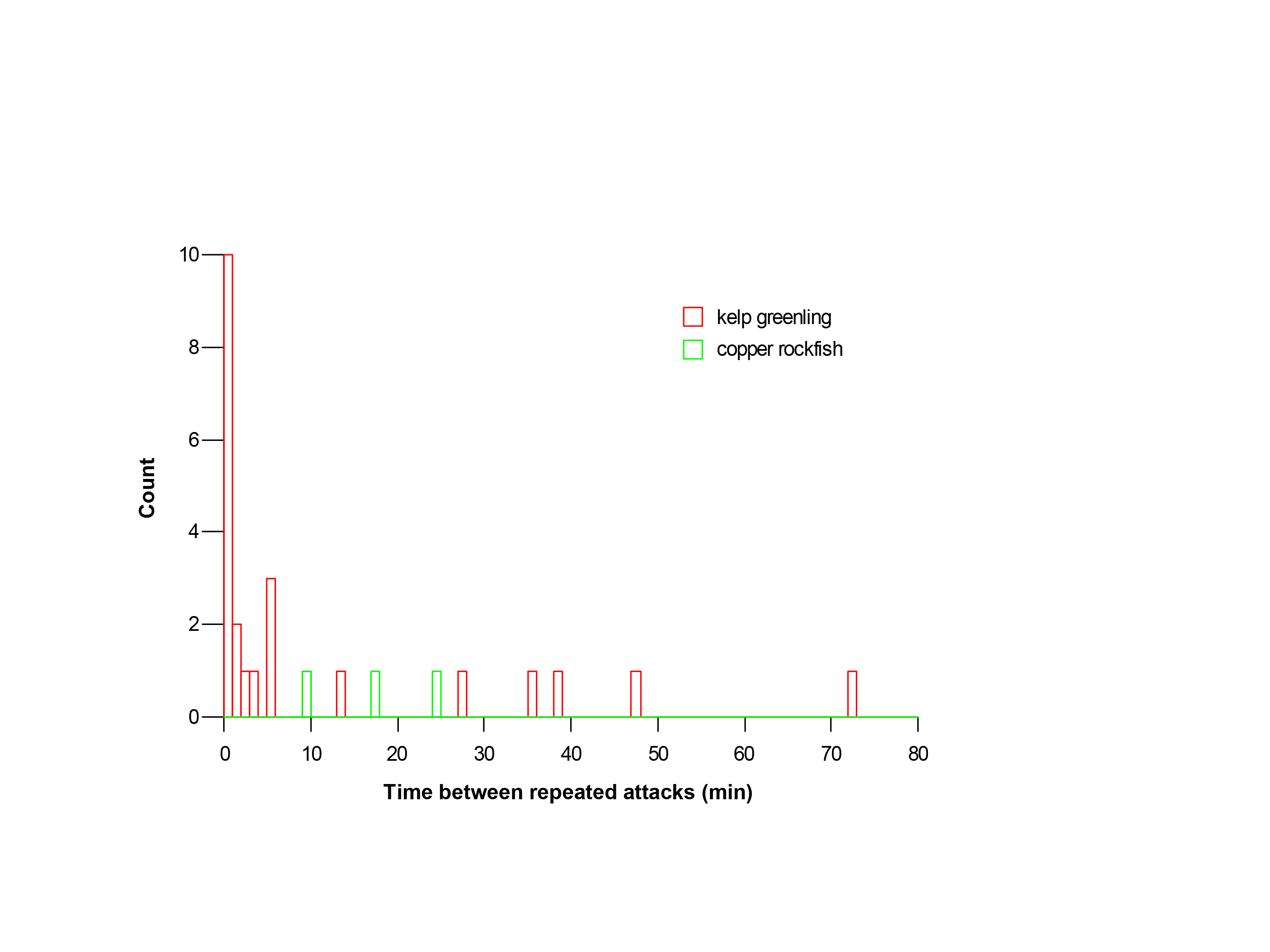

Supplement: Figure S1 — Distribution of time intervals (min) between repeated attacks conducted by kelp greenling or copper during the same of trial. Other species did not conduct repeated attacks. (TIF) [file pone.0040083.s001.tif]

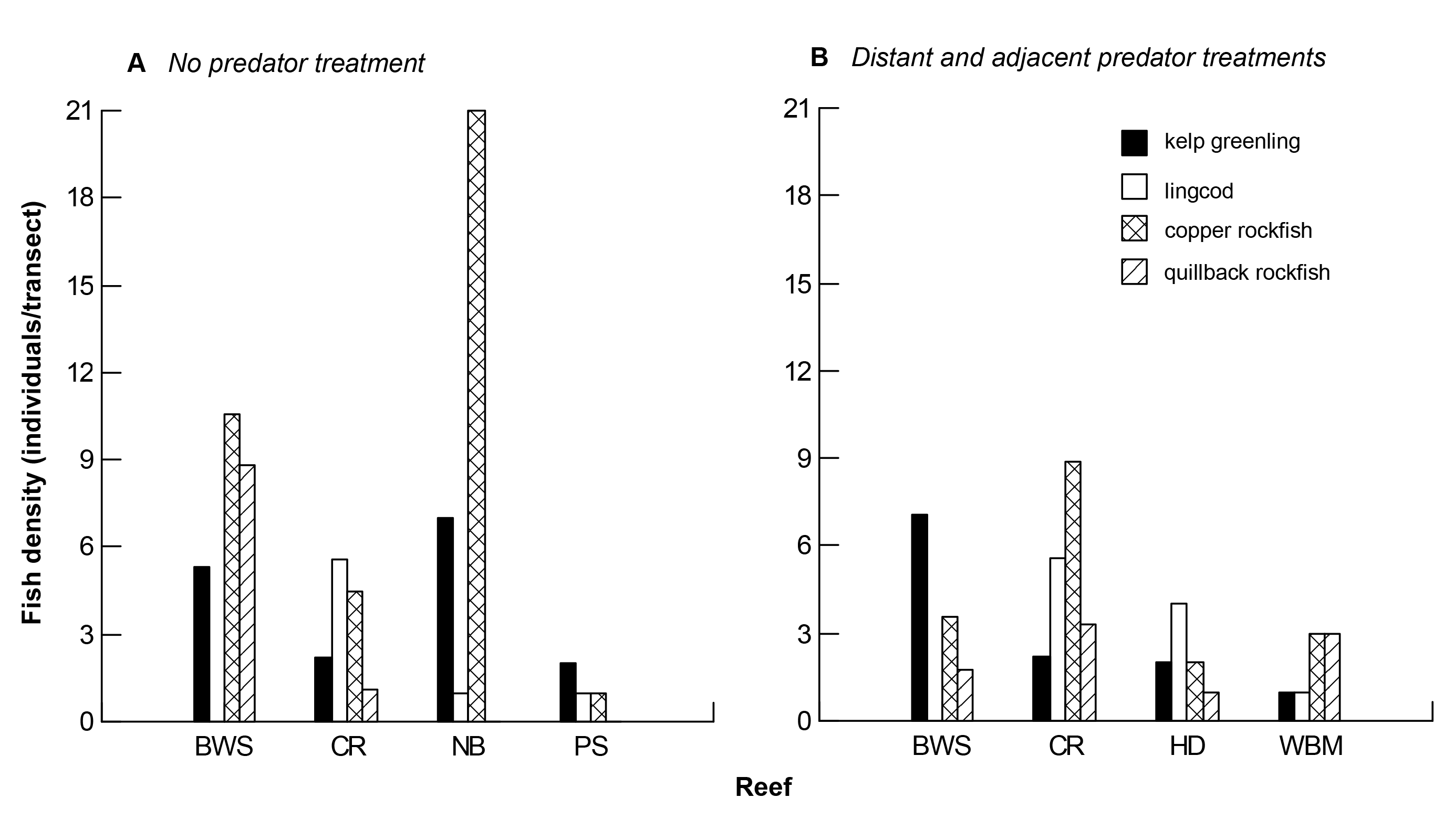

Supplement: Figure S2 — Fish densities estimated from counts of fish along 30 m×4 m transects conducted at the end of each experimental trial in 6 reefs of Howe Sound, British Columbia, October-December 2011. BWS and CR are the only reefs where both no predator and predator treatments occurred. (TIF) [file pone.0040083.s002.tif]
